# Polybenzimidazole Membrane Crosslinked with Epoxy-Containing Inorganic Networks for Organic Solvent Nanofiltration and Aqueous Nanofiltration under Extreme Basic Conditions

**DOI:** 10.3390/membranes12020140

**Published:** 2022-01-24

**Authors:** Jaewon Lee, Hyeonmin Yang, Tae-Hyun Bae

**Affiliations:** Department of Chemical and Biomolecular Engineering, Korea Advanced Institute of Science and Technology, Daejeon 34141, Korea; jaewon_lee@kaist.ac.kr (J.L.); asdfhjkl8998@kaist.ac.kr (H.Y.)

**Keywords:** polymer membrane, polybenzimidazole, organic-inorganic network, organic solvent resistant nanofiltration, alkaline resistant membrane

## Abstract

In this study, a novel polybenzimidazole (PBI)-based organic solvent nanofiltration (OSN) membrane possessing excellent stability under high pH condition was developed. To improve the chemical stability, the pristine PBI membrane was crosslinked with a silane precursor containing an epoxy end group. In detail, hydrolysis and condensation reaction of methoxysilane in the 3-glycidyloxypropyl trimethoxysilane (GPTMS) yields organic–inorganic networks within the PBI membrane structure. At the same time, the epoxy end groups on the organosiloxane network (Si–O–Si) reacted with amine groups of PBI to complete the crosslinking. The resulting crosslinked PBI membrane exhibited a good stability upon exposure to organic solvents and was not decomposed even in basic solution (pH 13). Our membrane showed an ethanol permeance of 27.74 LMHbar^−^^1^ together with a high eosin Y rejection of >90% under 10 bar operation pressure at room temperature. Furthermore, our PBI membrane was found to be operational even under an extremely basic condition, although the effective pore size was slightly enlarged due to the pore swelling effect. The results suggest that our membrane is a promising candidate for OSN application under basic conditions.

## 1. Introduction

Organic solvents are heavily utilized in the pharmaceutical and petrochemical industries as indispensable resources [1]. For instance, reaction mixtures used in pharmaceutical productions typically contain c.a. 85% of organic solvents [2]. Alcohols such as methanol, ethanol, and isopropanol are also required in pharmaceutical processes to obtain the desired products [3]. Thus, in the pharmaceutical and petrochemical industries, an efficient separation of organic solvents should be implemented not only to reduce energy consumption, but also to promote the recycling of solvents. In this context, organic solvent nanofiltration (OSN) or solvent-resistant nanofiltration has been emerging as a desired separation process due to several advantages over conventional processes such as low energy consumption and operation cost [4,5,6]. Modular design and small footprint, which are key advantages of membrane-based separation, are also highly beneficial in practical applications.

In general, OSN membranes typically have a molecular weight cut-off (MWCO) of 200–1000 Da [5]. To date, polymer materials are dominantly used in OSN membrane fabrication due to their processability and cost-effectiveness [7,8]. Indeed, polymeric OSN membranes could successfully demonstrate their potential for industrial applications [9,10]. To ensure the stability under organic solvent environments, membrane polymers should have a 3D network structure [11,12]. Thus, polymeric membranes are generally crosslinked via post-treatments using chemical agents or thermal energy [13]. For example, the polyaniline is known to be crosslinked by either chemical crosslinkers or thermal treatment [14].

The most well-known commercial OSN membranes are made up of polyimide (PI). In general, the PIs can be readily crosslinked with various diamines, leading to the formation of a 3D network structure [15,16]. However, such PI membranes cannot keep their stability under low and high pH conditions due to the vulnerability of the carbonyl group of the imide ring [17,18]. However, organic solvent separations under basic conditions are required in many practical applications such as cleaning process in food industry [19]. Hence, it is necessary to develop OSN membranes that are stable in organic solvents as well as at basic conditions.

Polybenzimidazole (PBI) has also been explored for OSN membrane fabrication due to its stability as well as the ability to be crosslinked [20,21,22]. PBI can be modified with chemical crosslinkers through amine groups in an imidazole ring. Xing et al. [23] fabricated a PBI OSN membrane crosslinked with glutaraldehyde and 1,2,7,8-diepoxyoctane. The resultant membranes exhibited high rejections for various dyes in ethanol and ethyl acetate. Valtcheva et al. [17] also reported a PBI membrane crosslinked with an aromatic alkyl halide, which showed a stable separation performance under low and high pH conditions. Furthermore, Wang et al. [24] demonstrated an excellent divalent anion selectivity of PBI membrane at high pH.

**Figure 1:**
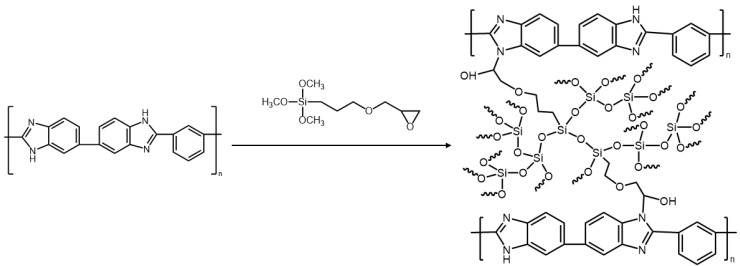
Chemical crosslinking of PBI with GPTMS.

Herein, we report a novel PBI membrane modified with 3-glycidyloxypropyl trimethoxysilane (GPTMS) with the aim to improve the chemical stability, even at harsh conditions. GPTMS was chosen due to the presence of an epoxy group, which can form strong primary bonding by the reaction with the imidazole ring in PBI. Such silane reagents can also form robust inorganic networks within the resulting membrane (Figure 1), leading to further enhancements in both mechanical strength and stability. Recently, Lim et al. [25] and Siddique et al. [26] incorporated the 3-aminopropyl trimethoxysilane into PI membranes and verified that the mechanical strength and chemical stability of the resulting membrane can be improved by the formation of an inorganic network structure. In this work, PBI membrane was fabricated by a non-solvent induced phase separation technique. After being crosslinked with GPTMS, our OSN membrane was tested to investigate both the molecular separation performance and chemical stability under a strong basic condition.

## 2. Materials and Methods

### 2.1. Materials

The Celazole^®^ S26 PBI (MW = 27,000 g mol^−1^) solution was obtained from PBI Performance Products Inc. (Tuscaloosa, AL, USA). The solution contains 26 wt% PBI and 1.5 wt% lithium chloride (stabilizer) in N,N-dimethylacetamide (DMAc). Non-woven polypropylene fabric (Novatexx 2471) was purchased from Freudenberg Filtration Technologies (Weinheim, Germany). All solvents such as DMAc (TCI, Chuo-ku, Japan), isopropanol (IPA, Daejung, Busan, Korea), acetonitrile (MeCN, Daejung), ethanol (EtOH, Daejung), N,N-dimethylformamide (DMF, Fisher, Al Khobar, Saudi Arabia), N-methyl-2-pyrrolidone (NMP, Alfa, Tewksbury, MA, USA), acetone (Daejung), and tetrahydrofuran (THF, Daejung) were used as received. GPTMS was purchased from Sigma (St. Louis, MO, USA). Rose Bengal (RB; 1017.6 g mol^−^^1^), methyl blue (MB; 799.8 g mol^−^^1^), and eosin Y (EY; 691.9 g mol^−^^1^) were obtained from Daejung, and tetracycline (TC; 444.4 g mol^−^^1^) was purchased from TCI. These were used as model solutes in OSN performance testing.

### 2.2. Fabrication of Crosslinked PBI Membranes

The Celazole^®^ S26 solution was diluted to 17 wt% PBI by adding DMAc and continuously stirred at room temperature to make a dope solution. After removing bubbles in the dope solution, membrane was cast on the non-woven fabric using a casting knife with a gap of 250 μm. The nascent membrane was then immersed in tap water at ambient temperature for phase inversion. Next, the membrane was soaked in IPA at least for one day to remove any residual solvents and impurities. For chemical crosslinking, the PBI membrane was immersed in the crosslinking solution comprising 2% (wt/vol) GPTMS in a 1:1 volume mixture of MeCN and DI water. Then, the solution was gradually heated and kept at 80 °C for 24 h. After the reaction, the resultant membrane was placed in IPA to remove unreacted agents.

### 2.3. Characterizations

Fourier transform infrared (FTIR) spectra of the modified and unmodified membranes were obtained using Nicolet iS50 (Thermo Fisher Scientific Instrument) with attenuated total reflection mode. X-ray photoelectron spectroscopy (XPS) was performed with Axis-Supra (Kratos) to analyze the elemental composition of the membrane surfaces. Field emission scanning electron microscope (FE-SEM) was employed to observe the surface and cross-section morphologies of the membranes. To prepare the sample for cross-sectional image, membranes were fractured in liquid nitrogen. Prior to the analysis, all samples were sputtered with osmium under an argon atmosphere. The images were taken under 3 kV. Energy dispersive X-ray (EDX) spectroscopy was utilized to examine a distribution of the Si element in the crosslinked PBI membrane.

To investigate the stability in organic solvent environments, the unmodified and modified membrane samples were immersed in DMF, NMP, and DMAc, respectively. Prior to the immersion test, membrane samples were cut into the same size, dried, and weighted. Then, all dried membrane pieces were soaked in 35 mL of the above-mentioned organic solvents contained in 50 mL vials. After five days, the membrane samples were collected from the solvents. The membrane samples were washed with DI water and dried under vacuum prior to measuring weight.

We also carried out permeation testing with several organic solvents such as DMF, acetone, and THF to verify the chemical stability and feasibility of our PBI membrane for OSN application. Filtration tests were performed at room temperature with a high-pressure stirred cell (Sterlitech HP4750) under dead-end mode. The operated pressure was set at 10 bar and the effective membrane area was 14.6 cm^2^.

### 2.4. OSN Performance Test

The OSN performance was examined using ethanol as the representative solvent under cross-flow conditions at ambient temperature. The effective membrane area of a permeation cell was 18.86 cm^2^ and the flow rate was kept at 0.6 L min^−^^1^ during the tests. The permeance of pure ethanol was measured at 10 bar of applied pressure at room temperature and the data were obtained every hour. A steady state was assumed when three consecutive flux values were recorded within a 5% margin. The test was repeated four times with fresh membrane samples to check the reproducibility. To evaluate the solute rejection properties, membranes were first compacted at 20 bar for 1 h in a cross-flow cell to rule out the compaction effect of fresh membrane. Then, the operation pressure inside the cross-flow cell was set to 10 bar. We confirmed that permeance and rejection values of the membranes were quite stable after compaction at the above-mentioned conditions. In order to study the separation performance of our membranes, dyes that are soluble in ethanol such as RB, MB, EY, and TC were utilized. Separation performance was reported from at least three membrane samples to verify the reproducibility.

The permeance can be calculated by Equation (1);

(1) $$P=\frac{V}{\mathrm{At}\Delta p}$$

where P is the permeance; A is the membrane area; t is the time; ∆p is the pressure drop; and V is the volume of the permeate.

The apparent rejection is calculated by the following Equation (2);

(2) $$R=\left(1-\frac{C_{p}}{C_{f}}\right)\times 100$$

where R is the rejection in percent, and $C_{f}$ and $C_{p}$ are the solute concentrations of feed and permeate, respectively.

To compare the mechanical stabilities of unmodified and modified membranes, a long-term filtration test with pure ethanol solvent was conducted under cross-flow mode at 10 bar operation pressure.

In order to evaluate the alkaline stability of the crosslinked PBI membrane, an aqueous RB solution at pH 13, which was adjusted by NaOH, was fed to the membrane cell. The operation conditions were set at the pressure of 10 bar at room temperature with the feed flow rate of 0.6 L min^−^^1^. Prior to testing, the membrane was operated with the dye aqueous solution feed at neutral conditions. After reaching the steady-state, a filtration of basic solution was performed for 24 h.

## 3. Results and Discussion

### 3.1. Characterizations of PBI Membranes

#### 3.1.1. FTIR and XPS

**Figure 2:**
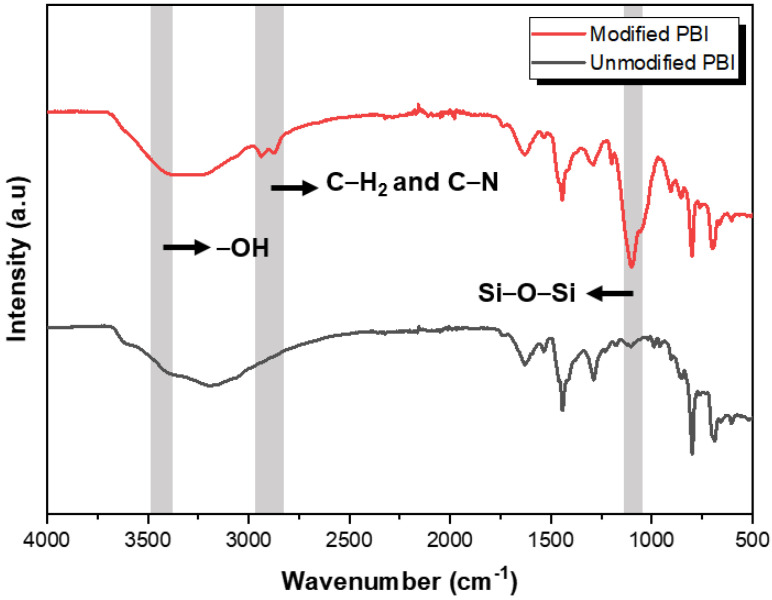
FTIR spectra of PBI membranes before and after crosslinking with GPTMS.

Figure 2 shows the FTIR spectra of both unmodified and modified PBI membranes. For both samples, an absorption band that appeared at 3190 cm^−1^ is ascribed to N–H stretching, and multiple bands in a broad range (at 1630, 1590, 1444, 1290, 800 cm^−1^) can be assigned to the benzene and imidazole rings as well as their conjugation [27,28]. Compared to pristine PBI, the characteristic band at 1099 cm^−1^ is shown in the spectrum of the modified PBI membrane, which can be attributed to the Si–O–Si bond. This implies that the siloxane network is successfully formed within the PBI membrane by the condensation reaction of GPTMS [29,30,31]. The intensity of the absorption band at 3428 cm^−1^ increased after the crosslinking reaction, indicating the opening of the epoxy ring and formation of the hydroxyl group (–OH) [32]. Two absorption bands appeared at 2873 and 2937 cm^−1^ corresponding to C–N and C–H bonds, respectively, indicating the successful crosslinking of PBI with GPTMS [17].

**Figure 3:**
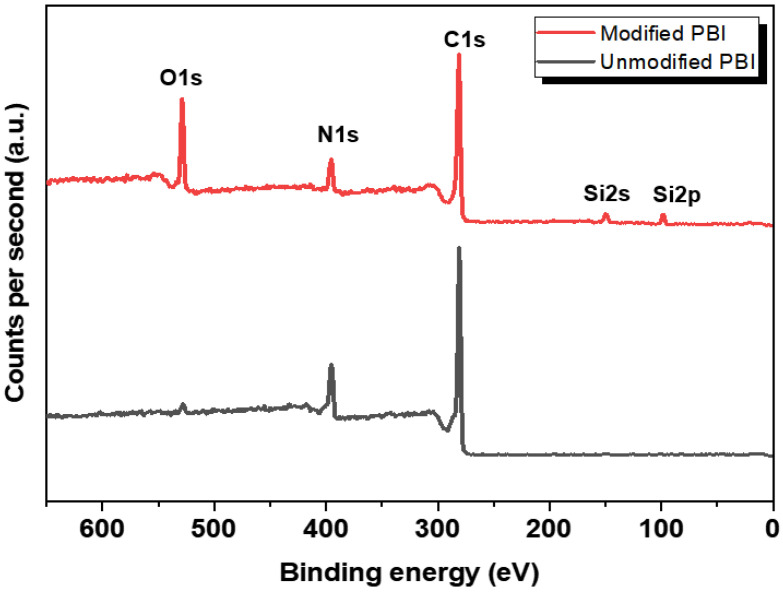
XPS spectra of the surfaces of both unmodified and modified membranes.

To further confirm the crosslinking of PBI, XPS analysis was also conducted. Figure 3 displays the full survey XPS spectra of both unmodified and modified PBI membranes. The elemental compositions at the membrane surfaces are also shown in Table 1. The oxygen content was found to increase after the modification, presumably due to the oxygen atom in the crosslinker. Moreover, silicon atoms, which do not exist in the pristine membrane, appeared in the crosslinked membrane as expected [25]. The oxygen to silicon ratio of the modified membrane was calculated to be 3.53, which is close to the theoretical oxygen to silicon ratio of the crosslinker. All these results may imply that the continuous Si–O–Si networks were formed in the PBI membrane during the modification reaction [26].

#### 3.1.2. Visual Observation with FE-SEM

**Figure 4:**
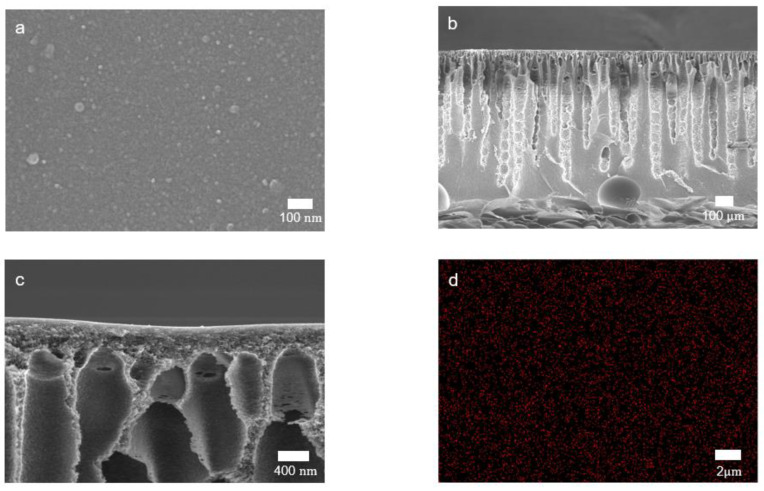
SEM images of crosslinked PBI membranes. (**a**) Surface and (**b**) cross-sectional images of overall morphology; (**c**) cross-sectional image of the skin layer; (**d**) EDX elemental mapping of silicone on the membrane surface.

The surface and cross-sectional images of the crosslinked PBI membrane are shown in Figure 4a–c. No appreciable voids or pores were observed at the membrane surface, indicating the formation of a highly dense skin layer. A typical asymmetric morphology, which is the result of non-solvent induced phase inversion, was observed in the cross-sectional image [33]. While the sponge-like structure with domains in the micrometer-range is shown in the dense skin layer, irregular finger-like macrovoids are formed in the sublayer. The sponge-like structure and macrovoids can provide the mechanical strength to withstand a high operation pressure and less-resistant mass transfer channels, respectively [12,20]. EDX mapping was also performed to investigate the distribution of silicon atoms on the crosslinked PBI membrane surface. As shown in Figure 4d, Si atoms were distributed homogeneously on the membrane surface as a result of the uniform reaction between PBI and GPTMS [25].

#### 3.1.3. Chemical Stability Test

In order to test the feasibility of our crosslinked PBI membrane for application in OSN, both unmodified and modified membranes were soaked in several polar aprotic solvents for five days. As the solvent resistance is a key property of OSN membrane, harsh organic solvents such as DMF, DMAc, and NMP were selected as testing solvents [34,35,36]. Weight loss was calculated by using the gel fraction formula, and photographic images of PBI membranes taken after the immersion are shown in Table 2. The pristine PBI membrane samples were completely dissolved immediately after immersion in all organic solvents [27]. Only the non-woven polypropylene fabric support was left while the color of the solvent was changed to yellow. On the other hand, neither visible change nor weight change was observed for all crosslinked PBI samples after the immersion in organic solvents for five days, as shown in Table 2. Through the successful chemical crosslinking, polymers might form three-dimensional networks possessing a high robustness and chemical stability. Overall, the immersion testing confirmed that the crosslinking reaction with GPTMS imparts an excellent solvent resistance to PBI.

**Figure 5:**
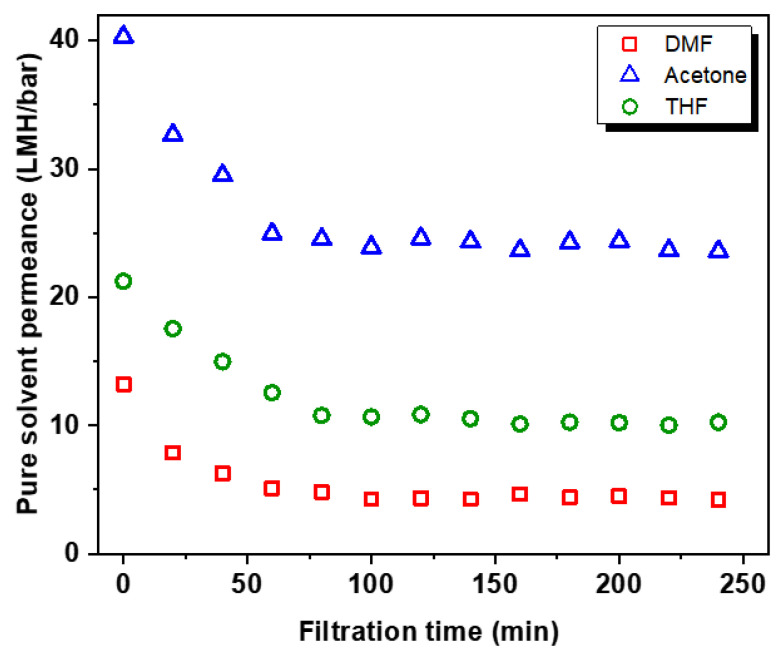
Initial permeation profiles of pure solvents including DMF, acetone, and THF through the crosslinked PBI membranes measured at 10 bar and room temperature. Unmodified PBI membrane was not testable due to its instability.

In addition to the immersion test, permeation testing with organic solvents such as DMF, acetone, and THF was also conducted to investigate the potential utility of our membranes in OSN application. Figure 5 shows the permeance profiles of three pure solvents over time that were measured under dead-end filtration mode at 10 bar. After the membrane compaction caused by a high operation pressure, the performance of the crosslinked PBI membrane was stabilized for all solvents [37]. In contrast, unmodified membranes were decomposed during the filtration test. The results imply that our crosslinked PBI membrane has a solvent resistance, and thus potential utility in OSN application.

### 3.2. Performance Test for OSN

#### 3.2.1. Performance under Ethanol Condition

**Figure 6:**
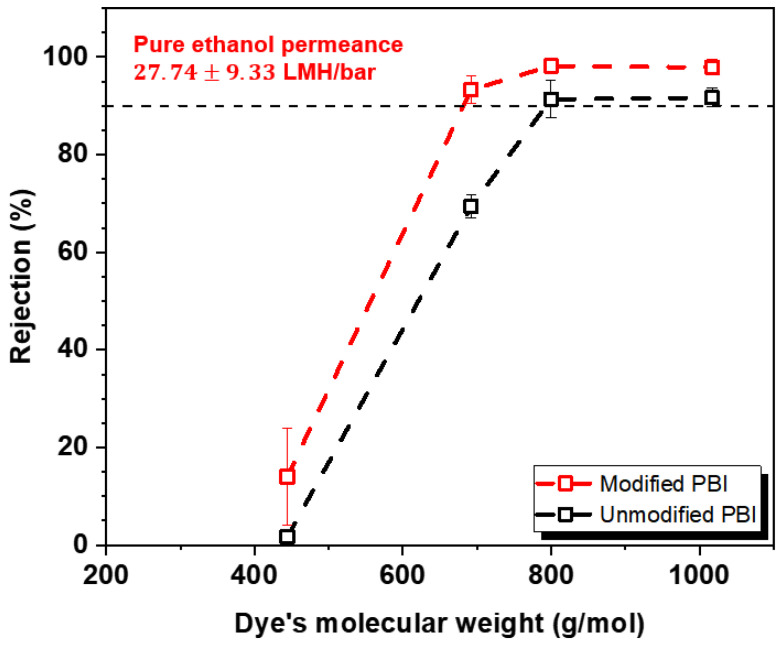
The rejection profiles of PBI-based membranes in an ethanol environment. Dye molecules with various molecular weights were dissolved in ethanol solution and used for the rejection test. Pure ethanol permeance of the crosslinked PBI membrane is also indicated in the figure.

Ethanol was chosen as the representative solvent because it has been extensively used in the pharmaceutical industry [38,39]. Several dyes with various molecular weights, namely, RB, MB, EY, and TC were employed as model solutes to evaluate the rejection properties of uncrosslinked and crosslinked PBI membranes. Figure 6 exhibits the OSN performance including the pure ethanol permeance and dye rejections measured at 10 bar. Our modified membrane showed the pure solvent permeance of 27.74 LMH bar^−1^. The modified membrane showed superior separation performance than the unmodified membrane. Our membrane displayed a sharp rejection profile, which is desired for an efficient molecular discrimination [40]. The uncrosslinked PBI membrane showed the rejection values of EY and TC at 69% and near 0%, respectively. In contrast, for the crosslinked PBI membrane, the rejections of three dye molecules with a molecular weight over 690 g/mol were found to be very high (>90%), while the rejection of TC was measured to be very low in the range of 4~24%. Overall, the MWCO value of the modified membrane decreased from 800 to 680 Da in an ethanol environment, indicating that the effective pore size of the PBI membrane slightly decreased after the crosslinking reaction. [39]. It is noteworthy that the OSN performance of the modified membrane is comparable to those of commercial OSN membranes as well as other membranes recently reported in the literature [23,27,38].

**Figure 7:**
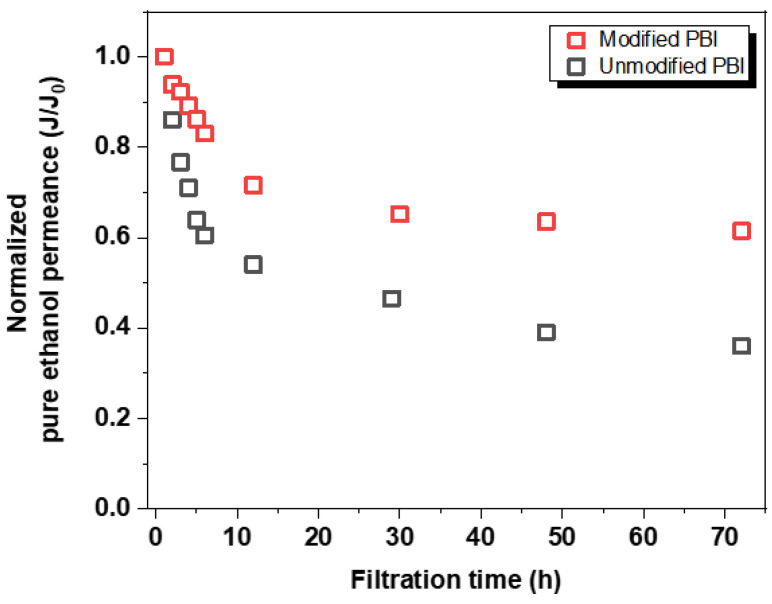
Long-term ethanol flux behavior of both unmodified and modified PBI membranes.

To investigate the durability of PBI membranes, we monitored the ethanol permeances of both unmodified and modified membranes over 72 h. Figure 7 shows the normalized flux of both PBI membranes over filtration time. For pristine PBI membrane, a sharp decrease (greater than 40%) in pure ethanol permeance was occurred within 6 h. In contrast, the crosslinked PBI membrane exhibited a relatively stable behavior. In fact, decreases in permeances observed for both PBI membranes are due to the compaction effect under the high operation pressure (10 bar) [41]. After 72 h of continuous operation, both modified and unmodified membranes reached steady-state normalized fluxes at 0.62 and 0.36, respectively. Such an excellent compaction resistance for crosslinked PBI membrane is ascribed to the formation of a 3D network structure as well as the presence of siloxane networks, which can reinforce the overall membrane structure [30]. It is worth noting that one of the major disadvantages of polymeric membranes is the flux decline over time due to the compaction effect [42]. However, the incorporation of the inorganic network into a polymeric membrane could allow the membrane to possess a long-term durability, as reported elsewhere [26]. Overall, our results indicate that our PBI membrane crosslinked with rigid siloxane networks had an outstanding mechanical strength, leading to a highly stable performance under a high operation pressure.

#### 3.2.2. Filtration Test under Basic Condition

**Figure 8:**
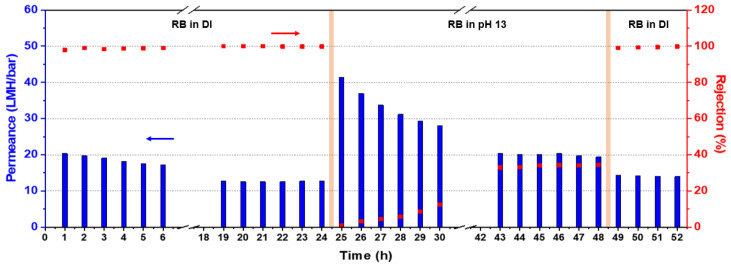
Water permeance and RB rejection profiles of the crosslinked PBI membrane at both neutral and pH 13 conditions.

The filtration of a basic aqueous solution was performed to validate the chemical stability of our crosslinked PBI membrane, and the results are shown in Figure 8. For the first 24 h, the permeance and rejection were measured under neutral condition, where our PBI membrane showed almost 100% rejection of RB. However, the permeance gradually decreased over time due to both the compaction, as mentioned in the previous section, and the deposition of RB molecules on the membrane surface, leading to an increase in mass transfer resistance. When the filtration time reached 24 h, the stable performance was observed. However, the separation performance was dramatically changed as soon as the feed solution was replaced with a basic aqueous solution at pH 13. Initially, the permeance increased to 40 LMH bar^−1^ and the rejection of RB decreased to 0%, which is possibly due to the pore swelling and the removal of RB molecules on the membrane surface [43]. Then, the performance was gradually stabilized at this new condition as time went on, although the permeance and rejection values were not similar to those in the neutral condition. Indeed, when the exposure time to the basic condition reached 24 h, the membrane showed the permeance of 20 LMH bar^−1^ and dye rejection of 34% in contrast to 13 LMH bar^−1^ and 100% rejection recorded at neutral condition.

**Figure 9:**
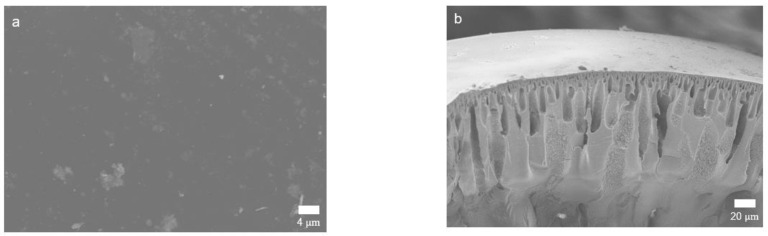
(**a**) Surface and (**b**) cross-sectional SEM images of the crosslinked PBI membrane after the filtration test at pH 13.

Most importantly, our PBI membrane was not decomposed under this extremely high pH solution, which implies that our membrane possesses an outstanding alkaline resistance. Hence, the separation performance was quickly retrieved when the filtration condition was switched to neutral. The permeance and RB rejection were recorded at 14 LMH bar^−1^ and 99.7%, respectively. The surface and cross-sectional images of our membrane were also taken using FE-SEM after conducting a filtration test at pH 13. As shown in Figure 9, there was no evident change in the crosslinked membrane structure compared to the pristine membrane (Figure 4). Overall, our results indicate that crosslinking of the PBI membrane with GPTMS, leading to the formation of inorganic siloxane networks within the resulting membrane, is an effective strategy to fabricate an alkaline-resistant OSN membrane.

## 4. Conclusions

In this work, we fabricated a novel PBI-based OSN membrane that not only has excellent separation performance, but also a strong alkaline resistance. It was found that crosslinking of the PBI membrane with GPTMS, allowing the formation of robust siloxane networks within the membrane, can improve both the chemical stability and compaction resistance. The crosslinked PBI membrane was not dissolved in harsh organic solvents such as DMF, NMP, and DMAc and showed a relatively stable permeance during a long-term operation under 10 bar operation pressure. Furthermore, our PBI membrane was found to be operational even under an extremely basic condition, although the effective pore size was slightly enlarged due to the pore swelling effect. Considering the ease of membrane fabrication and modification together with all of the desired properties above-mentioned, we expect that our PBI membrane can potentially be utilized in food and pharmaceutical industries where separations are often conducted in an organic solvent phase under basic conditions.

## Figures and Tables

**Table 1:** Elemental compositions of PBI membranes measured from the XPS analysis.

Sample	C1s (%)	N1s (%)	O1s (%)	Si2p (%)	O/Si Ratio
Unmodified PBI	79.81	17.74	2.45	-	-
Modified PBI	70.37	10.05	15.26	4.32	3.53

**Table 2:** Weight losses of PBI membrane samples after immersion in three organic solvents for five days. Photographic images of the membranes taken during the immersion test are also provided.

Membrane	Weight Loss (%)					
	DMAc		DMF		NMP	
Unmodified PBI	30 ± 2	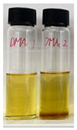	29 ± 1	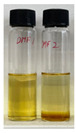	28 ± 0	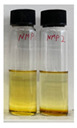
Modified PBI	0 ± 0	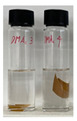	0 ± 0	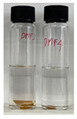	0 ± 0	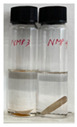

## Data Availability

Not applicable.
